# Electrochemical detection of *Toxocara canis* excretory-secretory antigens in children from rural communities in Esmeraldas Province, Ecuador: association between active infection and high eosinophilia

**DOI:** 10.1186/s13071-020-04113-2

**Published:** 2020-05-12

**Authors:** Francisco Morales-Yánez, Stanislav Trashin, Idalia Sariego, Clémentine Roucher, Linda Paredis, Martha Chico, Karolien De Wael, Serge Muyldermans, Philip Cooper, Katja Polman

**Affiliations:** 1 Laboratory of Cellular and Molecular Immunology, Vrije Universiteit Brussel, Brussels, Belgium; 2 Department of Biomedical Sciences, Unit of Medical Helminthology, Institute of Tropical Medicine, Antwerp, Belgium; 3 AXES Research Group, Department of Chemistry, University of Antwerp, Antwerp, Belgium; 4 Institute of Tropical Medicine “Pedro Kourí”, Havana, Cuba; 5 Fundación Ecuatoriana Para Investigación en Salud, Quito, Ecuador; 6 Facultad de Ciencias Médicas, de la Salud y la Vida, Universidad Internacional del Ecuador, Quito, Ecuador; 7 Institute of Infection and Immunity, St George’s University of London, London, UK; 8 Department of Health Sciences, Section Infectious Diseases, VU University Amsterdam, Amsterdam, The Netherlands

**Keywords:** Toxocara, Eosinophilia, Nanobodies, Electrochemical assay

## Abstract

**Background:**

The diagnosis of active *Toxocara canis* infections in humans is challenging. Larval stages of *T. canis* do not replicate in human tissues and disease may result from infection with a single *T. canis* larva. Recently, we developed a nanobody-based electrochemical magnetosensor assay with superior sensitivity to detect *T. canis* excretory-secretory (TES) antigens. Here, we evaluate the performance of the assay in children from an Ecuadorian birth cohort that followed children to five years of age.

**Methods:**

Samples were selected based on the presence of peripheral blood eosinophilia and relative eosinophil counts. The samples were analyzed by the nanobody-based electrochemical magnetosensor assay, which utilizes a bivalent biotinylated nanobody as capturing agent on the surface of streptavidin pre-coated paramagnetic beads. Detection was performed by a different nanobody chemically labelled with horseradish peroxidase.

**Results:**

Of 87 samples tested, 33 (38%) scored positive for TES antigen recognition by the electrochemical magnetosensor assay. The average concentration of TES antigen in serum was 2.1 ng/ml (SD = 1.1). The positive result in the electrochemical assay was associated with eosinophilia > 19% (*P* = 0.001). Parasitological data were available for 57 samples. There was no significant association between positivity by the electrochemical assay and the presence of other soil-transmitted helminth infections.

**Conclusions:**

Our nanobody-based electrochemical assay provides highly sensitive quantification of TES antigens in serum and has potential as a valuable tool for the diagnosis of active human toxocariasis.
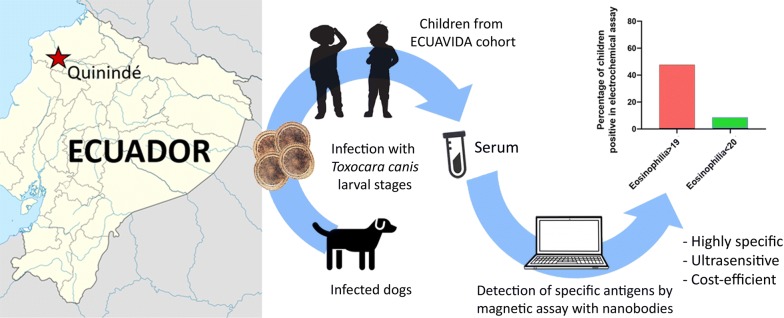

## Background

Human toxocariasis (HT) is an infection caused primarily by the roundworm *Toxocara canis* and to a lesser extent by *T. cati* [1]. It is a zoonosis with a worldwide distribution including temperate regions, although greater prevalence rates are observed in tropical areas [2–4]. Human infection occurs after the accidental ingestion of food or soil contaminated with eggs of the parasite. The larvae migrate to the lungs, liver, eyes and central nervous system of the host causing two disease variants: a compartmentalized form represented by ocular larvae migrans (OLM) and neurotoxocariasis [5], and a disseminated form represented by visceral larvae migrans (VLM), resulting from massive ingestion of embryonated eggs, and covert toxocariasis (CT) that is far more frequent than the other forms [5–7]. All the forms of the disease are frequently accompanied by eosinophilia [8].

Currently, the laboratory diagnosis of the disease is based on the detection of IgG antibodies (Ab) against the *T. canis* excretory-secretory (TES) antigens, a mix of highly glycosylated proteins released by migratory larvae into the tissues [9]. Limitations of the TES-based antibody detection (TES-Ab) ELISAs are low specificity [10, 11] and the inability to distinguish current from past infections. Anti-TES immunoglobulins may remain in the circulation for years after infection [12], limiting the usefulness of the test for monitoring response to treatment or the occurrence of active infections at a population level. Eosinophil counts in peripheral blood are often used to aid in the diagnosis of HT [1, 13, 14]. However, eosinophilia is associated with other helminth parasite infections or may be absent in active HT [8]. The heterogeneous clinical representation of the disease, combined with the lack of highly specific and sensitive diagnostic tools, makes the diagnosis of HT very challenging.

The detection of TES antigens has been proposed as an alternative to TES-Ab ELISAs with limited success to date [15–18]. Larval stages of *T. canis* release TES antigens into host tissues of which only a small fraction reaches the systemic circulation. Mouse models indicate that after the ingestion of 50 eggs, TES antigen is detectable in the circulation three days post-infection while anti-TES antibodies appear after three weeks [19]. This indicates a limited time during the course of the infection when TES antigens are not complexed with immunoglobulins.

Recently, we developed a diagnostic system based on single domain antigen-binding fragments (nanobodies, Nbs) from camel heavy-chain antibodies with a highly sensitive electrochemical readout. This system employs Nbs as specific TES antigen binders, which were able to detect TES in serum from mice infected with *T. canis* eggs. Due to their small size, Nbs are able to recognize cryptic epitopes on their cognate antigen [20]. This approach has been demonstrated to provide high specificity with no cross-reactivity with antigens of other helminths [21, 22] and a sensitivity in the low picogram range [22]. Here, we evaluate the performance of the electrochemical magnetosensor assay in samples of children in an Ecuadorian birth cohort [23]. We investigated the association of positivity for TES antigen recognition in the Nb-based electrochemical assay with peripheral blood eosinophilia, and with positive *Toxocara* serology (as assessed by TES-Ab ELISA). Additionally, we evaluated potential cross-reactivity of the electrochemical assay in samples from children infected with other soil-transmitted helminth (STH) infections.

## Methods

### Production of TES antigens

TES was produced as described by de Savigny [24]. The final material was dialyzed in phosphate-buffered saline (PBS) pH 7.4 and lyophilized in separate batches according to the source larvae. The antigens were reconstituted in Milli-Q water and the concentration was estimated in triplicate by UV spectrophotometry (Nanodrop), (OD_280nm_ of 1 corresponds to 1 mg/ml).

### Construction of the immune library and Nbs selection

Detailed protocols for library construction and Nbs selection are available elsewhere [20, 25]. *In vivo* biotinylated nanobodies 2TCE49 and monovalent nanobodies 1TCE39 [21] chemically coupled to horseradish peroxidase were used as reagents to capture TES.

### Serum immunocomplex dissociation (ICD)

Serum samples were subjected to immune complex dissociation (ICD) to dissociate possible endogenous antibodies that can potentially block the Nb binding sites of TES antigens [18]. Briefly, 250 µl of serum was mixed with 250 µl of 100% glycerol and thoroughly vortexed. The mixture was diluted in 500 µl of 1.0 M ethylenediaminetetraacetic acid (EDTA) (pH 8) and heated in boiling water for 10 min. The samples were immediately centrifuged at maximum speed (19000×*g*) for 10 min and the supernatant was collected for further analysis.

### Electrochemical assay

The electrochemical assay was performed according to a protocol developed by our group [22]. The assay was performed on the surface of streptavidin pre-coated magnetic beads (50 µg/sample) (Dynabeads™ M-280; Thermo Fisher Scientific, Waltham, USA). After each step the beads were collected in a magnetic rack for 2 min and washed 3 times. First, we covered the beads with bivalent biotinylated Nbs 2TCE49 (2 µg/ml) in PBS Tween 20 0.05% (PBS-T20) to obtain a coverage of 2 µg of Nbs per mg of beads. Then, 100 µl of the supernatant from the immunocomplex dissociation was added to the beads together with Nb 1TCE39 coupled to HRP at a final concentration of 10 ng/ml. The samples were incubated at room temperature for 15 min in agitation. After that, the supernatant was discarded and each sample was diluted in 15 µl of 0.5 mM citrate-acetate buffer (35 mM sodium acetate, 50 mM citric acid, pH 5.0), and injected in 60 µl of reading buffer [(0.5 mM of 3,3’,5,5’-Tetramethylbenzidine (TMB), 2 mM H_2_O_2_ diluted in 0.5 mM citrate buffer)] placed on the surface of a screen-printed electrode (SPE; Metrohm DropSens, Oviedo, Spain). A neodymium magnet was attached to the underside of the electrode for accumulating the signal of the reaction. The readout of the reaction was performed *via* amperometry. The electrode was connected to a potentiostat EmStat Blue (PalmSens, Houten, The Netherlands) and the reaction was registered for 180 s with a potential of 0.0 V. The average of the last 20 s was calculated. Negative AB male human serum extracted from plasma (Sigma-Aldrich, St. Louis, USA) and subjected to exactly the same protocol of immunocomplex dissociation was used as a negative control. The limit of detection of the assay (LOD) was calculated as the average of at least five blanks. A standard curve was constructed in negative serum (Sigma-Aldrich) spiked with TES in 10-fold dilutions and treated with the ICD protocol. Additionally, we constructed a standard curve in PBS-T20 spiked with TES. Extrapolation of the concentration of TES in serum was performed using a linear model and taking into consideration sample dilution after ICD. Samples were run in duplicate.

### TES-Ab ELISA

To detect specific anti-TES IgG in ELISA (TES Ab-ELISA), we used a commercial ELISA for the diagnosis of human toxocariasis (Bordier Affinity, Crissier, Switzerland), according to the manufacturer’s instructions. The reaction was read in an ELISA spectrophotometer equipped with a filter for the 405 nm wavelength. All samples were run in duplicate.

### Patients and samples

A total of 87 plasma samples from the ECUAVIDA birth cohort were used in this analysis. The ECUAVIDA birth cohort study was designed to study the effects of early life exposure to STH parasites on the development of allergy [23]. The study was performed in the tropical rural district of Quinindé, Esmeraldas Province, Ecuador. Stool and serum samples were collected between 2007 and 2012 when the children reached 13, 24 and 36 months of age. One study group consisted of 22 samples from children with eosinophilia < 20% of total white blood count (WBC): 11 samples from children aged 13 months and 11 samples from children aged 24 months. The second group consisted of 65 samples from children with eosinophilia > 19% of total WBC: 7 samples from children aged 13 months, 18 samples from children aged 24 months and 40 samples from children aged 36 months.

### Clinical and laboratory data

Children were followed up routinely at 13, 24 and 36 months of age; a complete clinical evaluation was carried out by a study physician, a blood and stool sample were collected, and a questionnaire was administered to the child’s mother to collect information on any health problems during the previous 12 months. Whole blood and differential counts were estimated using venous blood following standard laboratory procedures [23] and plasma was separated by centrifugation (19000×*g*) and stored at − 30 °C until use. Stool samples were examined using a combination of microscopic methods that were conducted on all samples with sufficient quantity: direct saline smear; modified kato-katz (quantification of soil-transmitted helminth eggs); and formol-ethyl acetate concentration methods. The detailed parasitological procedures for stool analysis are available elsewhere [26]. Modified kato-katz and concentration methods were performed on duplicate slides. A stool sample was considered positive if STH helminth eggs or larvae were detected by at least one of the three methods.

### Data analysis

Chi-square test was used to determine if there was an association (i) between positivity in the TES-Ab ELISA and eosinophilia groups (> 19% and < 20%), and (ii) between positivity in the electrochemical assay and positivity in the TES-Ab ELISA in either group of eosinophilia (> 19% and < 20%). Fisher’s exact test was used to determine if there was an association (i) between positivity in the electrochemical assay and eosinophilia groups (> 19% and < 20%); (ii) between the presence of helminths and positivity in the electrochemical assay; and (iii) between the presence of helminths and positivity in the TES-Ab ELISA. Results were considered significant when the *P*-value was < 0.05. All data were entered into Excel, and SPSS version 15.0 and Prisma GraphPad 8.0. were used for statistical analysis.

## Results

### Electrochemical assay

**Fig. 1:**
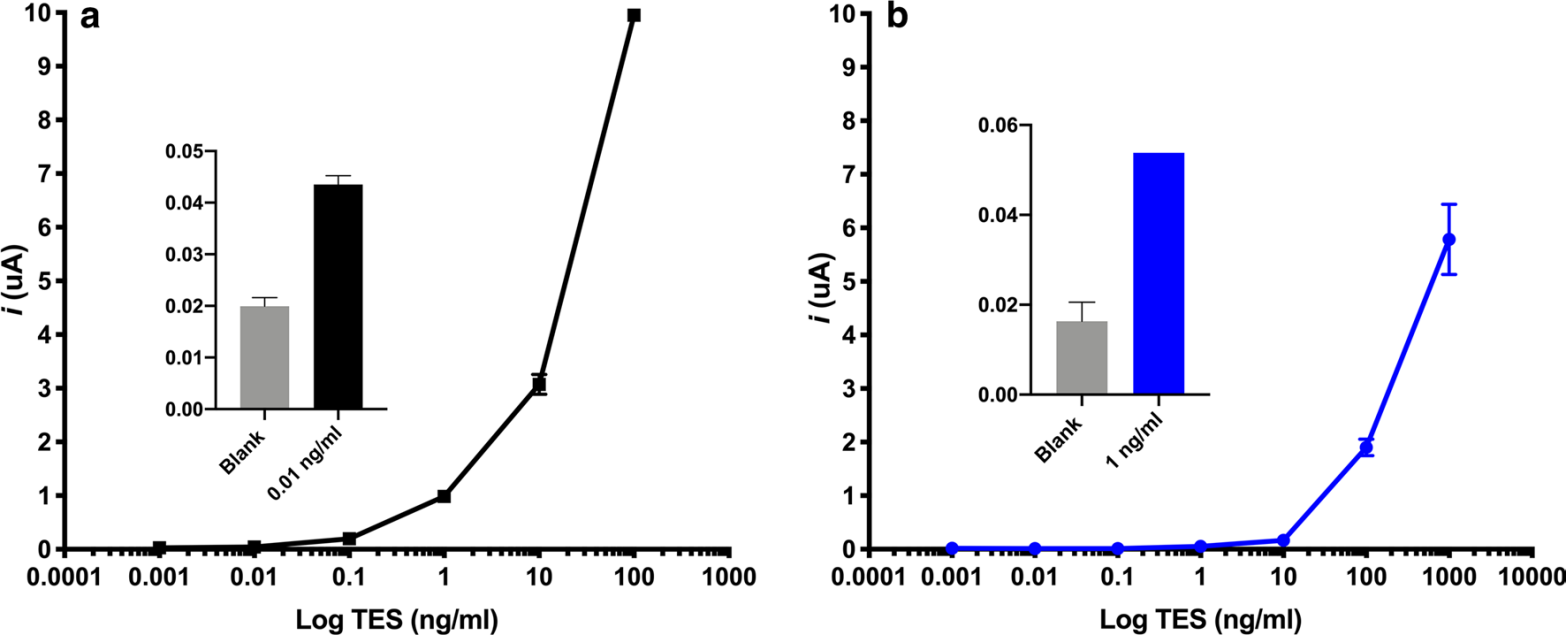
TES detection standard curves. **a** PBS-T20 spiked with TES. **b** Serum spiked with TES after ICD

**Fig. 2:**
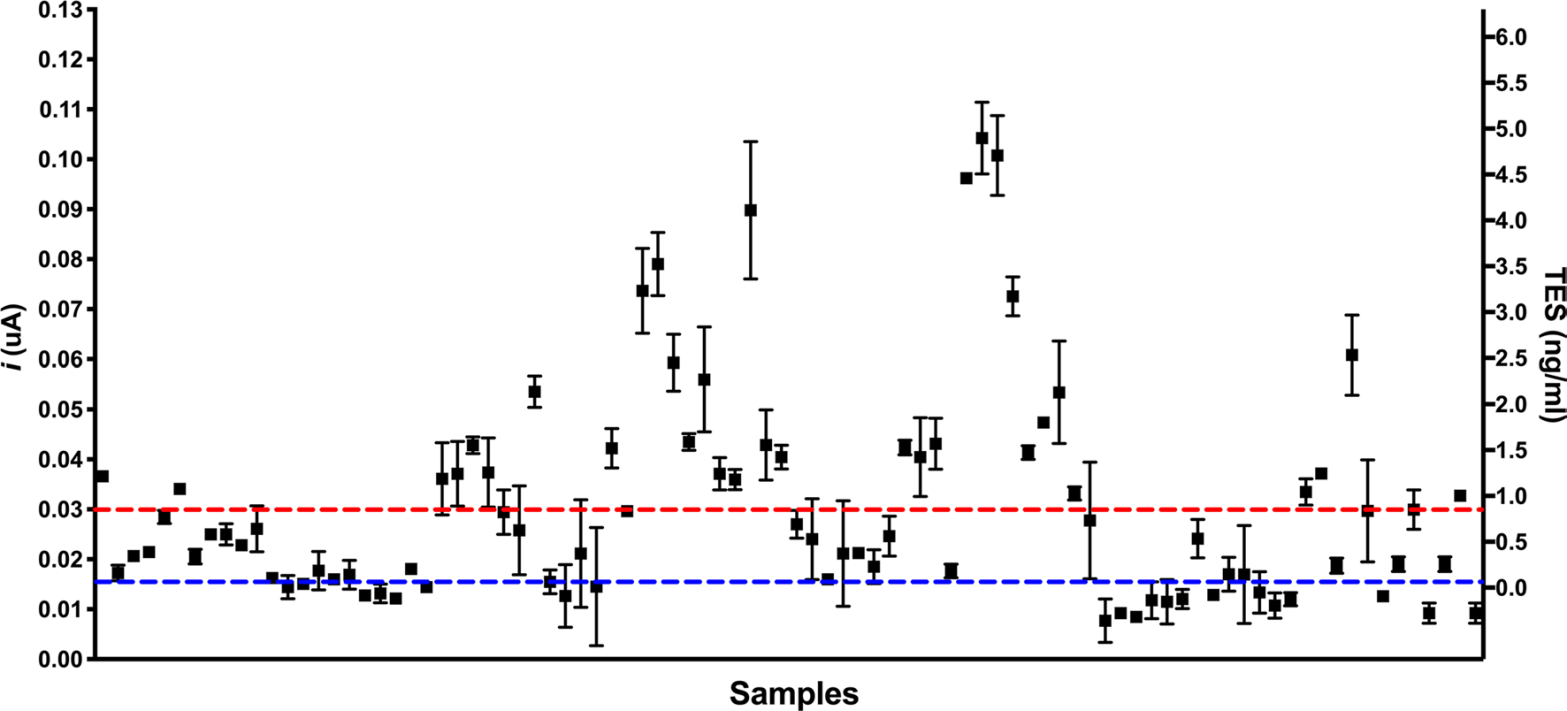
Plot of the full dataset showing the amperometric reading and the equivalent concentrations of TES antigen. The blue dotted line represents the blank of the assay (estimated as the average of 6 different reactions in negative serum without TES and subjected to ICD). Red dotted line is the LOD of the assay, 0.9 ng/ml. The variation in each point represents the absolute reading number of each measurement. Only the measurements that were higher than the LOD in both measurements were considered as positive

Standard curves in serum and PBS-T20 spiked with TES antigen and subjected to immunocomplex dissociation (ICD) antigen showed a LOD of 0.01 ng/ml and 0.9 ng/ml, respectively (Fig. 1). In total, 38% (33/87) of the serum samples were positive in the electrochemical assay (Fig. 2). The average concentration of TES antigen in the serum of positive samples was 2.1 ng/ml (SD = 1.1).

### Role of eosinophilia in *T. canis* infections

**Fig. 3:**
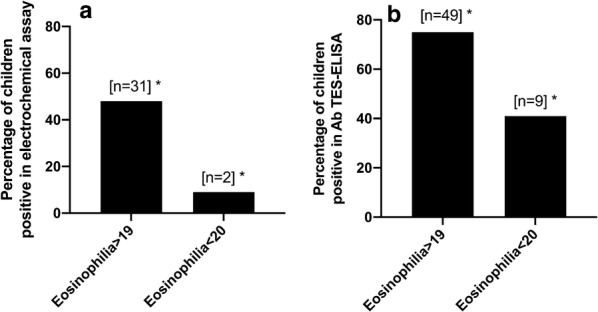
**a** Percentage of positives by electrochemistry in children with high (> 19%) and low (< 20%) eosinophilia. **b** Percentage of positives by Ab TES-ELISA in children with high (> 19%) and low (< 20%) eosinophilia. Asterisks indicate significant association (*P* < 0.05)

Out of 87 samples, 38% (33/87) were positive by electrochemical analysis and 67% (58/87) by TES-Ab ELISA. Only 9% (2/22) of the samples from the group with eosinophilia < 20% were positive with the electrochemical assay, while 48% (31/65) were positive in the group with eosinophilia > 19% (Fig. 3a). For TES-Ab ELISA, these percentages were 41% (9/22) and 75% (49/65), respectively (Fig. 3b). Eosinophilia was significantly associated with positivity in the electrochemical assay (OR: 9.12, 95% CI: 2.20–41.25, *P* = 0.001) as well as in the TES-Ab ELISA (*χ*^2^ = 8.79, *df*  = 1, *P* = 0.003). In the group with eosinophilia > 19%, 50% (24/48) of the samples were positive in the TES-Ab ELISA and electrochemistry, while other 50% (24/48) were positive in the TES-Ab ELISA and negative in electrochemistry. In the group with eosinophilia < 20%, we found that only 11% (1/9) were positive in the TES-Ab ELISA and electrochemistry, and 89% (8/9) were positive in the TES-Ab ELISA and negative in electrochemistry. No significant association was found between electrochemical assay and TES-Ab ELISA positivity in either of the group of eosinophilia > 19% (*χ*^2^ = 1.09, *df * = 1, *P* = 0.26) or < 20% (OR: 1.5, 95% CI: 0.16–12.71, *P* > 0.99).

### Cross-reactivity with other helminth parasites

**Fig. 4:**
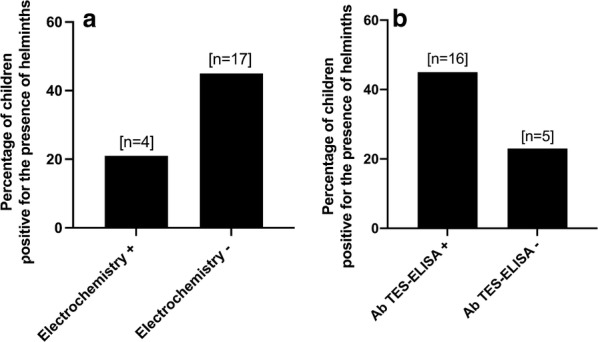
**a** Percentage of positives by electrochemistry in children with and without helminths (other than *T. canis*). **b** Percentage of positives by TES-Ab ELISA in children with and without other helminth infections. No significant association was observed between the percentage of helminth-positive and a positive outcome in either of the tests

Parasitological data were available for 57 samples. Helminth infections were found in 19% (4/19) of the samples that scored positive in electrochemistry (Fig. 4a). Two were positive for *A. lumbricoides* and two for *Trichuris trichiura*. Of the samples that scored negative in electrochemistry, 45% (17/38) were positive for the presence of helminths. For TES-Ab ELISA (Fig. 4b), these percentages were 44% (16/36) for *A. lumbricoides* and 24% (5/21) for *T. trichiura*. There was no significant association between helminth infections and positive outcomes in the electrochemical assay (OR = 0.33, 95% CI = 0.11–1.18, *P* = 0.14) or TES-Ab ELISA (OR = 2.56, 95% CI = 0.79–7.68, *P* = 0.16). The distribution of helminth infections by parasite is presented in Additional file 1: Figure S1.

## Discussion

### TES quantification

In the past, several attempts to detect TES antigen in clinical samples have been performed with limited success. A two-site antigen ELISA with the monoclonal antibody Tcn-2 was the first attempt to identify active HT infections [15, 26]. This assay had a LOD of 20 ng/ml and a sensitivity of 68, 10 and 29% in VLM, inactive infections (patients that were positive in TES-Ab ELISA and with a normal eosinophil count) and OLM, respectively. Other immunoassays showed a LOD of 78 ng/ml [17], 4 ng/ml [27] and 5 ng/ml [16]. The most recent attempt to detect TES antigen in clinical samples reported a LOD of 0.440 ng/ml [18]. These assays estimated the LOD by a standard curve constructed in PBS-Tween 20 as diluent. In contrast to the previously published studies on TES antigens detection, we used ‘negative’ serum spiked with TES and treated it with the ICD procedure before the TES detection, i.e. we applied exactly the same procedure as for the real samples to properly estimate the realistic LOD of the analysis. It rendered the LOD of 0.9 ng/ml and an average concentration of TES in positive samples of 2.1 ng/ml (SD = 1.1).

### Active HT is associated with eosinophilia

Eosinophilia is a common feature in helminth infections [28]. For HT, Gillespie et al. [26] reported eosinophilia > 0.4 × 10^9^/l in 28 patients with VLM; of these, 19 scored positive in TES antigen detection using a sandwich ELISA with the monoclonal Ab Tcn-2. In addition, the association between seropositivity (by TES-Ab ELISA) and eosinophilia has been extensively documented [1, 10, 13, 14, 29–33]. These data are in line with our findings. We found a significant association between eosinophilia > 19% of total WBC and a positive outcome by the electrochemical assay as well as by TES-Ab ELISA. Importantly, the samples used in the present study had eosinophilia > 19%, which is substantially higher than the traditional cut-off of 5% of total WBC count. Thus, very high eosinophilia could be a reliable, but non-specific, indicator of active HT. The usefulness of eosinophilia in the diagnosis of HT is limited since it is not a specific indicator of *T. canis* infection, and high levels of eosinophils can be found in other helminth infections, atopic and allergic diseases and adverse drug reactions [34].

Active *T. canis* infections are frequently diagnosed by the combination of a positive TES-Ab ELISA accompanied by eosinophilia [35]. In contrast, the electrochemical assay can be used as a single parameter to identify active infections. In our study, we found that half of the children with eosinophilia > 19% and positive by TES-Ab ELISA were also positive by the electrochemical assay. In contrast, only 11% of children with eosinophilia < 20% and positive by TES-Ab ELISA were positive by the electrochemical assay. These findings illustrate an important limitation of a TES-Ab ELISA positive result combined with high eosinophilia as the criteria to diagnose HT, which is that positive TES-Ab ELISA in the presence of high eosinophilia can in general occur and cannot certainly indicate active infections.

### No indication of cross-reactivity with other helminth parasites

The detection of TES in serum by ELISA has been associated with high cross-reactivity to other helminth parasites such as *Ascaris lumbricoides*, *Trichinella spiralis*, *Schistosoma* spp. and *Fasciola hepatica* [15, 26]. Cross-reactivity with other parasites is a problem, particularly in rural settings where polyparasitism is frequent [10, 36]. In our study, we did not find a significant association between positivity in the electrochemical assay and other STH infections. In fact, we found more helminth infections in children that scored negative by electrochemistry than in those that scored positive. This highlights the specificity of our test. The four children that had a positive outcome in the electrochemical test and were helminth positive, most likely had mixed infections. Due to the difficulty of defining truly negative controls for HT in endemic areas with available diagnostic methods, the determination of the specificity of TES antigen detection is difficult. Moreover, the small sample size in the present study complicates a precise interpretation of the results.

A major issue on the diagnosis of HT by the TES Ab-ELISA is the lack of specificity, particularly in countries where polyparasitism is prevalent. Western blot (WB) of serum against TES has been demonstrated to be more specific in combination with ELISA for the diagnosis of HT [37]. However, it requires equipment that is not always available in the field, and the procedure is more laborious, particularly for large sets of samples. While the latter aspect limits the application of WB for the diagnosis of HT, future studies should include this additional test, if conditions allow.

## Conclusions

The electrochemical assay was able to identify active HT infections in a group of 87 children from remote communities in the province of Esmeraldas in Ecuador. This is, to our knowledge, the most accurate quantification of TES in human serum samples reported so far. As the electrochemical assay provides evidence for the presence of *Toxocara* larvae, it has the potential to become a powerful instrument for the diagnosis of active HT infections. Further validation of the assay with a larger number of clinically and epidemiologically well-defined samples is needed.

## Supplementary information

**Additional file 1: Figure S1.** Frequencies of helminth infections in the samples analyzed.

## Data Availability

The datasets used and/or analyzed during the present study are available from the corresponding author upon reasonable request.

## Abbreviations

Ab: Antibodies
CT: Covert toxocariasis
EDTA: Ethylenediaminetetraacetic acid
HT: Human toxocariasis
ICD: Immunocomplex dissociation
IgG: Immunoglobulin G
LOD: Limit of detection
Nbs: Nanobodies Nbs
OLM: Ocular larva migrans
PBS: Phosphate-buffered saline
PBS-T20: PBS Tween 20 0.05%
SD: Standard deviation
STH: Soil-transmitted helminth
TES: *Toxocara canis* excretory-secretory antigens
TES-Ab ELISA: TES-based antibody detection
TMB: 3,3′,5,5′-tetramethylbenzidine
VLM: Visceral larva migrans
WB: Western blot
WBC: White blood count
