# Supplementary figures and images for: Electrochemical detection of Toxocara canis excretory-secretory antigens in children from rural communities in Esmeraldas Province, Ecuador: association between active infection and high eosinophilia
Source: Parasit Vectors. 2020 May 12;13:245. doi: 10.1186/s13071-020-04113-2 (PMC7216625; doi:10.1186/s13071-020-04113-2)

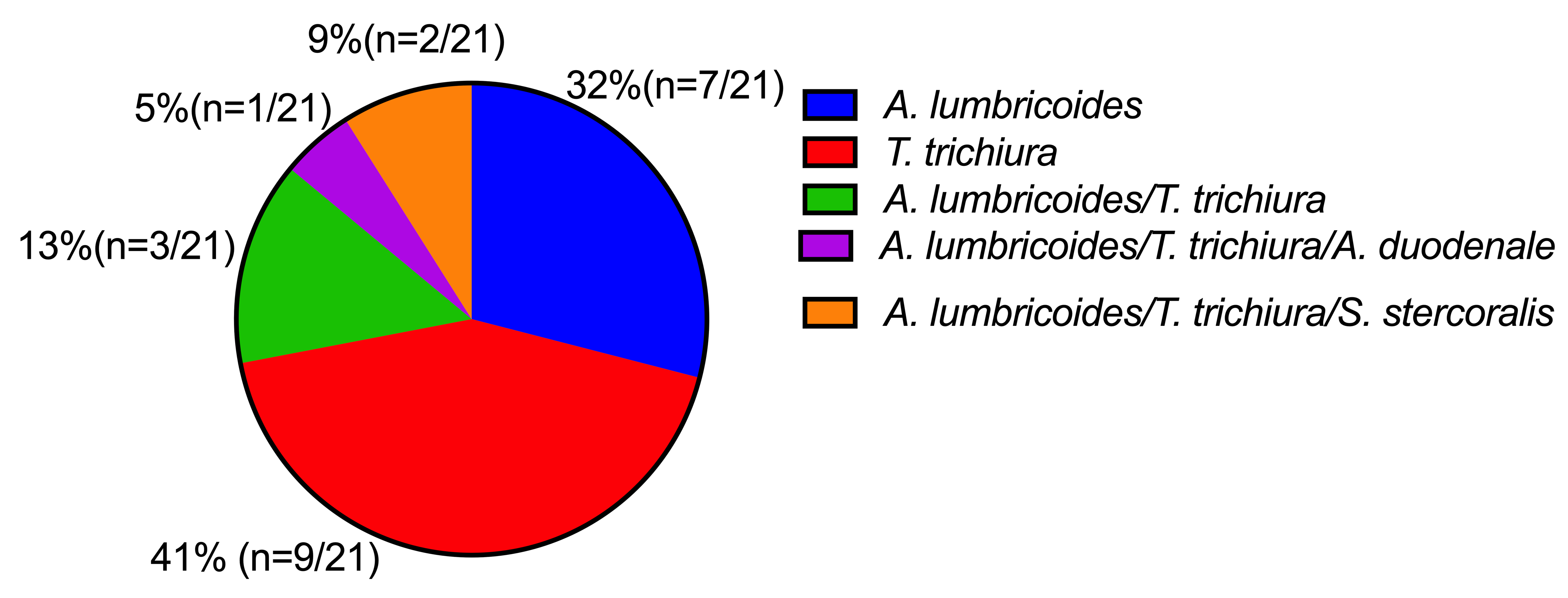

Supplement: Supplementary file 1 — Additional file 1: Figure S1. Frequencies of helminth infections in the samples analyzed. [file 13071_2020_4113_MOESM1_ESM.tiff]
